# Body image of men and women with congenital heart disease over a 15 years observational period

**DOI:** 10.1038/s41598-025-87097-2

**Published:** 2025-02-05

**Authors:** Siegfried Geyer, Claudia Dellas, Elmar Brähler, Johannes Beller

**Affiliations:** 1 Department of Medical Sociology, Medizinische Hochschule Hannover, Carl-Neuberg-Strasse 1, Hannover, 30625 Germany; 2 Department of Pediatric Cardiology, Intensive Care Medicine and Neonatology, University Medical Center, Göttingen, Germany; 3 Department of Medical Psychology and Medical Sociology, Department of Psychosomatic Medicine and Psychotherapy, Universitätsklinikum Leipzig, Leipzig and University Medical Center Mainz, Mainz, Germany

**Keywords:** Congenital heart disease, Body image, Panel study, Repeated measurement, Comparative study, Psychology, Cardiology

## Abstract

Most studies devoted to the psychological consequences of congenital heart disease (CHD) have dealt with consequences in terms of psychopathology. We wanted to consider two specific aspects of body image, “Rejecting body evaluation” and “Vital body dynamics”. We examined body image of CHD-patients as compared with the general population, the stability of body image of patients over time, and the relationship of body image with disease severity. The study combined a longitudinal (panel-) and a case-control design. The findings were based on a long-term study of CHD-patients with two surveys about 15 years apart with *N* = 244 who participated in both. The control group consisted of the same number of cases matched by age, gender and education drawn from a national survey designed to examine body image in the general population. More men than women were classified into the group with severe CHD. Body image differences between CHD-patients and controls were found only in men, but not in women, and they emerged primarily in terms of vitality. The two dimensions of body image turned out as moderately to strongly stable over time, relationships between disease severity and body image emerged only for perceived vitality. Contrary to expectation, effects of age and sex were largely absent. The body image of men turned out to be more affected by congenital heart disease than women. Body image is not stable, but it is changing with increasing age, and disease severity is affecting body image only in terms of the perception of physical performance.

## Introduction

Due to surgical advancements and longitudinal medical care, most patients (more than 90%) with congenital heart disease (CHD) now survive into adulthood although the rates are declining with increasing severity^1–4^. In the last decades in particular the rate of survival of patients with complex malformations has increased from 33.5% in 1996/97 to 49.9% in 2014/15^5^, but these patients continue to be highly burdened by concomitants such as associated complaints and comorbidity^6^. Some studies have tackled the chronic disease burden from a psychiatric perspective^7^, but psychopathological descriptions may not be appropriate and useful for describing the situation of patients with congenital heart disease. In an earlier study the proportion of CHD-patients with pathological anxiety and depression was reported to be high, but the findings were of limited validity due to a small sample of *N* = 22^8^. A recent German study reported elevated anxiety and depression scores in patients with congenital heart disease who rejected their disease, or if psychological concomitants determined their whole life^9^. In another study psychopathology scores of patients with CHD were significantly higher than those of reference groups^10^. A Dutch longitudinal study depicted psychological states of patients with a Dutch personality inventory and a scale assessing quality of life^11^ and compared patients with population-based standard values. In two measurements performed 10 years apart, patients had higher scores on self-esteem and a higher satisfaction with life than it was expected based on standard scores. Patients also reported more and increasing CHD-related physical restrictions.

In a more specific way, psychological consequences of CHD can be tackled by considering appearance and perceived physical performance as part of body image. Only a small number of studies have addressed body image. In recent studies appearance had been examined as related to the postoperative scar that may impair patients’ everyday life and functioning^12–15^. In addition, physical performance may be related to disease severity and increasingly perceptible as strength of residual symptoms or as increasing comorbidity. Physical limitations and comorbidity may also increase with patients’ age^2,16^.

Against the backdrop of the research literature quoted above we decided to adopt body image as the main psychological construct. It is to be understood as a multidimensional construct, because it depicts both negative as well as positive aspects. This makes it possible to depict negative and positive aspects of body image simultaneously.

In our study we wanted to consider body image of adult women and men with congenital heart disease by examining.

- Whether the body image of men and women with congenital heart disease differed from a general population sample.
- Whether body image of male and female patients with congenital heart disease was stable over 15 years.
- Whether congenital heart disease of differing degrees of severity were associated with body image, in the same study sample as assessed about 15 years apart.

## Methods

### Patient sample with congenital heart disease

The data used in the following analyses were drawn from two surveys of men and women with congenital heart disease. All patients were operated and continuously treated at the University Medical Centre of the University of Göttingen, Germany. The first survey was conducted in 2003/2004, the follow-up took place between 2017 and 2019. The study covered the whole range of congenital malformations, a long follow-up period and combined medical, psychological and sociodemographic data. Inclusion criteria of the first study were age (17 to 45 years), and individuals with mental impairments and syndromes associated with mental impairments were excluded. Patients who had undergone surgery of congenital heart disease at the University Medical Centre Göttingen (Germany) were invited to participate and provided informed consent. All assessments were performed as personal interviews, and the standardized instruments were completed while an interviewer was present. Follow-up was possible because in the pediatric cardiology department of this university hospital CHD-patients are continuously cared for after having reached adolescence.

Examinations included medical histories, medical examinations as well as laboratory and spiroergometric measures to obtain information on cardiac functioning. Detailed information on the complete study setting were published earlier^17,18^. The severity of congenital heart defects was classified according to type of surgery^19^ as curative, reparative, or palliative. Having undergone curative surgery, patients have no or minor residual symptoms, resulting in the absence or minor impairment. After reparative surgery, heart functions can be established close to normal or with residual symptoms. Palliative surgery can establish heart functions only incompletely, and patients have permanent impairments and continuously perceptible symptoms.

### General population sample as control group data

The cross-sectional general population sample was drawn from a survey institute on behalf of the University of Leipzig and interviewed by means of face-to-face interviews. The FKB-20 was completed by the respondents themselves in presence of an interviewer. As the survey covered a broader age range than our study, we used only respondents between 17 and 59 years with a sample size of *N* = 1513. This covered the whole age range of the first and the follow-up assessment of our patient population. A detailed account of the general population sample the data were drawn from was published earlier^20^.

### Body image as assessed by the body image questionnaire FKB-20

Body image was assessed using the Body Image Questionnaire FKB-20^21^. It is a self-report instrument developed for assessing the way how men and women are perceiving their body in non-clinical settings. The second purpose is to assess disturbances of body awareness and body image in clinical samples^22^. The instrument was tested for use in clinical settings^22^ and beyond and validated with data from a general population survey with random sampling of respondents^20^. The main construct is divided into two sub-dimensions, “Rejecting body evaluation” and “Vital body dynamics”. The first one depicts a negative attitude towards one’s own physical appearance, lack of well-being, and an attitude that the body fails to meet demands considered as normal. It is to be understood as the negative aspect of body image. The second dimension depicts positive aspects of body image, particularly feelings of vitality, strength, and pleasurable physical activity. The FKB-20 consists of 20 items with scores ranging from 1 to 5. Higher scores on “Rejecting body evaluation” are indicating higher rejection, and higher scores on “Vital body dynamics” are indicating higher perception of vitality. Thus, in empirical terms the scores of the subscales should be negatively correlated if their substantive interpretations are in line. The score of each of the two dimensions is composed of the sum of single item scores, and the scoring range of each scale may vary between 10 and 50.

The number of body image scales is relatively small^23^, and when our study was planned in 2001, only two were available in German language. We finally preferred the FKB-20 because the second instrument^24^ has 52 items instead of only 20, it is thus less economic in a study with a large patient sample. The FKB-20 also turned out to be more understandable in a cognitive test with patients that was performed during the planning phase of the study. The major strength of the FKB-20 finally emerged from the availability of general population data^20^ what made it possible to perform comparisons beyond the clinical setting.

### Matching cases and controls, and statistical analyses

All statistical analyses were performed using STATA 16MP^25^. For the first research question concerning comparisons of body image between men and women with congenital heart disease and the general population a 1:1 multivariate matching was performed. Age (in years), sex (men, women) and education (no degree, 8/9 years, 10 years, 12/13 years of school education, school education not yet finished) were used as criteria for assigning controls to patients. Body image differs between women and men, it should change with age, and differences had also been reported for educational level^20^. For each survey wave a separate group of controls had to be drawn due to different age ranges of the two subsequent waves.

### Comparisons with the general population

For drawing respondents from the general population and for matching cases and controls from the SOEP the supplementary ado-module CCMATCH was used^26^. Against the backdrop of multivariate matching, difference tests could be applied. The distributions of the two dimensions of body image turned out as deviating from the normal distribution, thus requiring to apply nonparametric tests. Finally, median tests and the Kolmogorov-Smirnov-Test were used. The median test applied is based on a χ²-statistic and gives the probability of an error that the distributions of two samples are different. The Kolmogorov-Smirnov-Test tests the two-sample- equality of distributions in two lines: The first one tests that the values of the first group (controls from the general population) are smaller than those of the second (patients with congenital heart disease). The second line tests the hypothesis that the values of the first group (controls from the general population) are larger than those of the group of patients with congenital heart disease^27^.

### Stability of body image over time

**Fig. 1:**
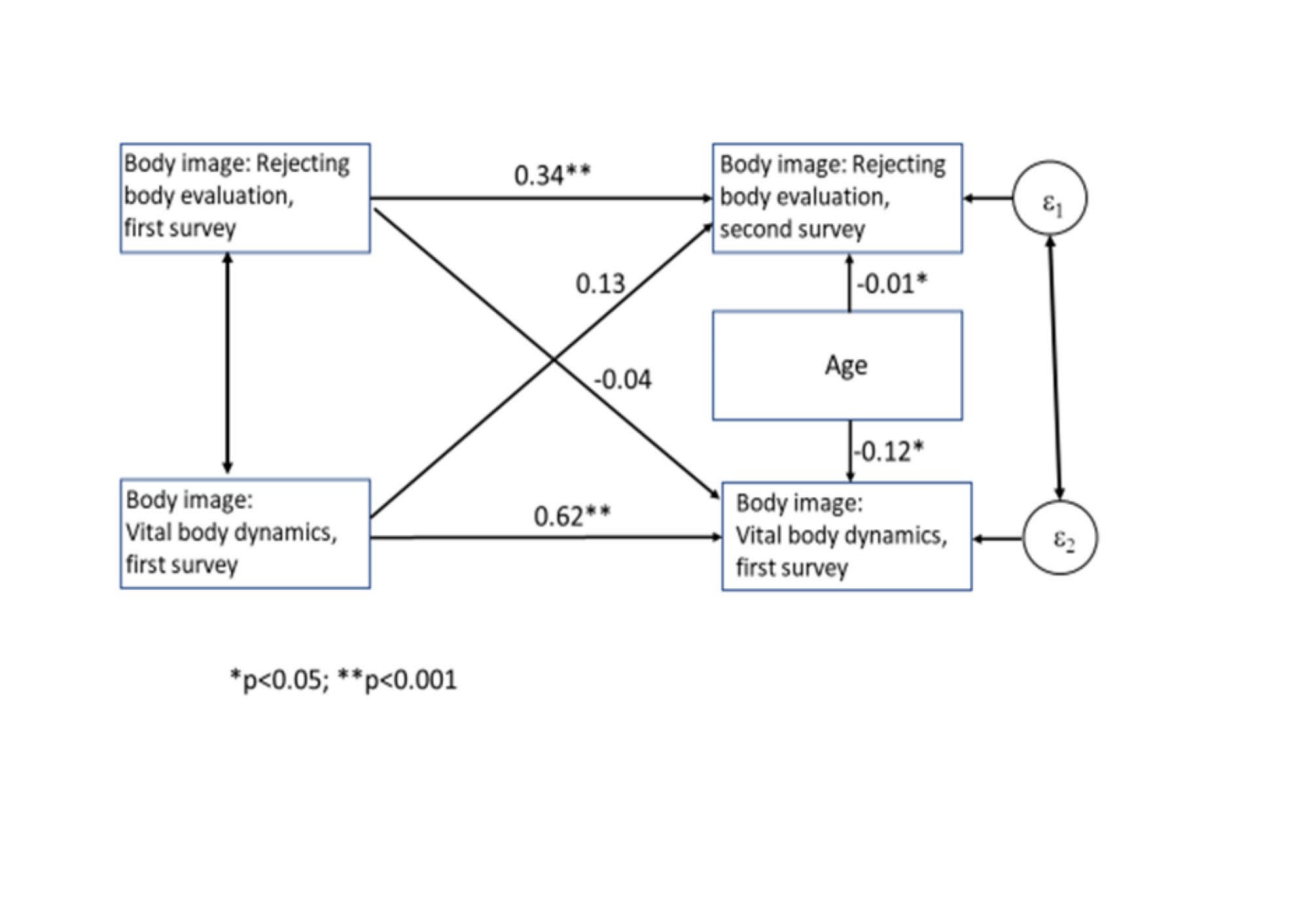
Men with congenital heart disease: Cross-lagged panel analysis for stability of the two body image dimensions.

**Fig. 2:**
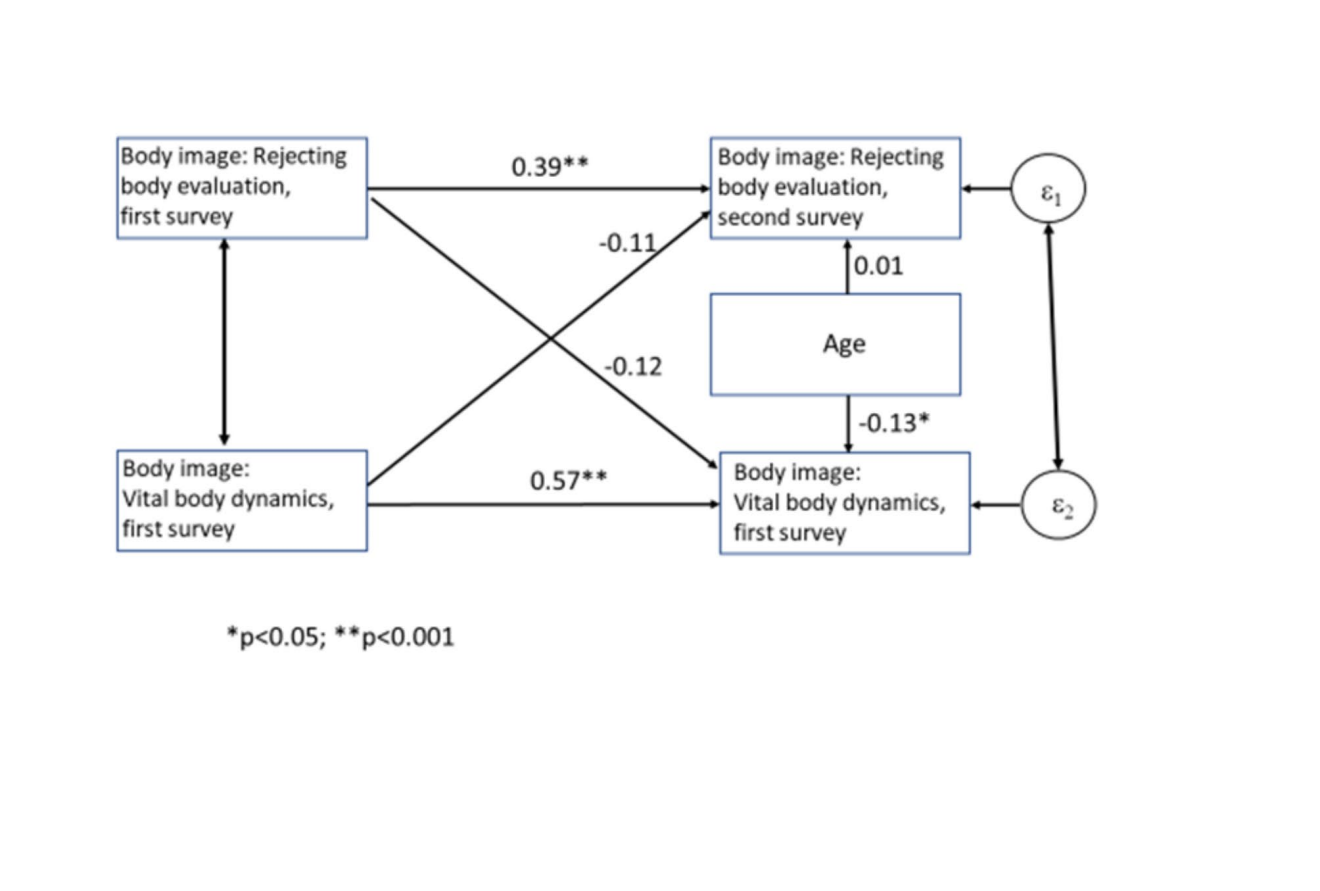
Women with congenital heart disease: Cross-lagged panel analysis for stability of the two body image dimensions.

The cross-lagged panel analyses were performed using the structural equation (SEM-) module of STATA 16^25^. A cross-lagged panel model was used in order to examine the stability of the two dimensions of body image over time, and as body image was demonstrated to differ between men and women^28^, stratified analyses were performed. The following paths were considered (see Figs. 1, 2) by using only the patient data:

1. All analyses were performed separately for men and for women.
2. Each FKB-20- dimension of the first survey wave was used as an exogeneous variable.
3. Stability coefficients were estimated as the main part of the analysis for each dimension of body image by permitting correlated residuals.
4. Effects of one dimension of body image to the second over survey waves were estimated.

### Disease severity and body image

Relationships between disease severity and body image were analyzed by means of ordinary least squares (OLS-) regression by considering each survey wave as well as men and women separately, thus resulting in four separate analyses. As tests for normal distribution revealed that the two FKB-20 dimensions are not distributed normally. Thus, analyses were based on 200 bootstrap-samples, i.e., analyses were repeated by drawing 200 subsamples with replacement^29^ in order to obtain confidence intervals based on normally distributed data.

## Results

Details on the 15-year development of the study sample with congenital heart disease (CHD) with information on panel attrition were published in two earlier papers^17,30^. When starting the study in 2004, 820 patients with CHD had ever been operated at the University Medical Centre. *N* = 698 patients remained after having excluded individuals with mental retardation and complex disabilities. After having excluded deceased patients and those whose addresses were unknown, 527 potential participants remained. After 121 refusals and 92 non-responders, 360 patients with complete social data made up the study population^18^. The starting sample of the present study with complete data of men and women who participated in both surveys comprised *N* = 244 individuals with a mean age of M = 25.73 (Sd = 8,63) years at the first and M = 39.80 (Sd = 8.59) years at the second survey. The mean age at the first surgery was 7.0 (Sd = 7.2) years^18^.

The rank-order correlations between both dimensions of body image were *r*=-0.52 for the first and *r*=-0.39 for the second survey. This result indicates that both dimensions are correlated, but most of the variance of the two scales is separate and below the magnitude indicating multicollinearity. It has to be kept in mind that higher scores on “rejection” are negatively and higher scores on “vitality” are positively connotated. The basic frequencies are depicted in Table 1. We refrained from including the arithmetic means of the two body image dimensions because they turned out as deviating from the normal distribution.

### Matching

The multivariate matching by age (in years), sex (men, women) and education (no degree, 8/9 years, 10 years, 12/13 years of school education, school education not yet finished) for the first assessment was successful for 239 out of 244 patients, for the second one 241 out of 244 matched cases were matched successfully. The analyses on stability of body image were run with matched cases only. As the number of patients with missing was small, it was not possible to perform sensitivity analyses for comparing cases with complete and incomplete data.

**Table 1:** Basic distributions of the variables used in the following analyses for patients with congenital heart disease (CHD), education refers to the second survey.

Type of surgery by sex (n/%)				
		Women (%)	Men (%)	Total (%)
First survey & Follow- up	Curative	25/24.5	19/13.4	44/18.0
	Reparative	68/66.7	96/67.6	164/67.2
	Palliative	9/8.8	27/19.0	36/14.8
	Total	102/ 41.8	142/ 58.2	244/ 100

Body image, Median						
	Rejecting body evaluation (Md)			Vital body dynamics (Md)		
Survey	Men	Women	Total	Men	Women	Total
1st CHD Controls	19 15	18 17	18 17	38 40	37 37	37 38
2nd CHD Controls	19 16	18 18	18 17	35 38	34 36	34 37

Cardiac functioning, measured by maximum oxygen uptake in % of reference values: Mean, SE, N of cases with valid data				
	Women	Men		Total
First survey	82.55/ 19.34 N = 90	69.91/ 14.77 N = 128		82.54/ 19.34 N = 218
Follow- up	90.44/ 22.21 N = 64	79.10/ 22.21 N = 97		83.60/ 22.39 N = 161
	School education, first survey (%)		School education, second survey	
No degree	4/ 1.67%		7/2.9%	
8/9 years	49/20.50		42/17.43%	
10 years	76/31.80		77/31.95%	
12/13 yrs	59/ 24.69		115/47.72%	
Not yet finished	51/21.34		0	

#### Body image- comparisons between CHD-patients and the general population sample (table 2)

The test of median differences of both dimensions of body image between cases and controls are statistically significant for men for the first survey for men. These findings are reproduced for the second survey for both dimensions of body image (Table 2).

**Table 2:** Tests for median differences and Kolmogorov-Smirnov-tests for FKB-20-differences in men and women based on data from the two surveys.

FKB-20 dimension	Sex	Median differences	Kolmogorov-Smirnov test
(1) Rejecting body evaluation, first survey	Men	χ² (1) = 10.80; *p* < 0.001	Controls D = 0.28; *p* < 0.01 CHD D=-0.20; *p* = 0.96
(2) Rejecting body evaluation, first survey	Women	χ² (1) = 0.53; *p* = 0.47	Controls D = 0.07; *p* = 0.53 CHD D=−0.08; *p* = 0.42
(3) Vital body dynamics, first survey	Men	χ² (1) = 8,78; *p* < 0.01	Controls D = 0.02; *p* = 0.96 CHD D=−0.22; *p* < 0.01
(4) Vital body dynamics, first survey	Women	χ² (1) = 0.28; *p* = 0.60	Controls D = 0.04; *p* = 0.84 CHD D=−0.12; *p* = 0.27
(5) Rejecting body evaluation, second survey	Men	χ² (1) = 9.16; *p* < 0.01	Controls D = 0.23; *p* < 0.01 CHD D= −0.01; *p* = 0.99
(6) Rejecting body evaluation, second survey	Women	χ² (1) = 0.92; *p* = 0.34	Controls D = 0.07; *p* = 0.49 CHD D=−0.07; *p* = 0.87
(7) Vital body dynamics, second survey	Men	χ² (1) = 8.65; *p* < 0.01	Controls D = 0.02; *p* = 0.96 CHD D=−0.24; *p* < 0.01
(8) Vital body dynamics, second survey	Women	χ² (1) = 4.94; *p* = 0.04	Controls D = 0.03; *p* = 0.89 CHD D=−0.17; *p* = 0.01

The Kolmogorov-Smirnov-tests are examining the direction of group differences directly. For the first survey in the first line of analysis 1 it was tested whether an unfavorable perception of one’s body among male controls is lower than among CHD-patients, and the test supports this assumption. In the second line of analysis 1 it was tested whether body rejection among male controls was higher than among CHD-patients, and this test failed to become statistically significant. The two lines of analysis 2 on group differences in women all tests failed to become statistically significant. Analysis 3 dealt with the perceived vitality of one’s body with higher scores are indicating a more favorable outlook. The respective test indicates that the score of controls are lower than those of CHD-patients. In contrast, the second test indicates that the control group score was higher than that of CHD-patients. For women (analysis 4) both difference tests again failed to become statistically significant.

The tests performed with data of the second survey the direction of findings replicated those of the first survey (analyses 5 to 7), albeit analysis 8 indicates group differences also for women to the detriment of patients with CHD.

As a supplementary analysis it was examined whether the distributions of the three categories of disease severity were differing between men and women. Based on a cross-tabulation of both variables it turned out that disease severity was not evenly distributed by sex (χ^2^(2) = 8.26; *p* = 0.02). This gender difference will be reconsidered in the regression analyses presented in Table 3.

#### Stability of body image over time: cross-lagged panel analyses (Figs. 1 and 2)

Separate power analyses were performed for men and for women to make sure that the case numbers of the two subpopulations were sufficient. Assuming a medium effect size of at least β = 0.12, a power level of 0.80, an error level of *p* = 0.05, a minimum sample size of *N* = 94 required. This was fulfilled for the male (*N* = 142) as well as for the female study population (*N* = 102).

Figures 1, 2 are depicting the relationships between the two dimensions of body image over time, and age. Rejecting body evaluation has decreased over time, and the effect of the first to the second surveys is statistically significant for men and for women, but standardized regression effects of 0.39 and 0.34 do not indicate high degrees of stability. In contrast, vital body dynamics turned out as more stable over time. The effects of the other dimension of body image are all small and not statistically significant. Finally, it was examined whether the effects of the stability coefficients of “rejecting body evaluation” and “vital body dynamics” differ with respect to their stability over time. The test reveals that this was the case in the male (χ²(1) = 4.33; *p* = 0.04) as well as in the female subpopulation (χ²(1) = 3.87; *p* = 0.05).

As supplementary analysis it was examined whether the scores of the two dimensions of body image differed over time, i.e., between the two measurements by performing T-tests for mean differences > 0. For rejecting body evaluation, the mean score differences (M_1men_ = 20.11; 95% CI 18.82–21.40/ M_2men_ = 19.46; 95% CI: 18.26–20.67) were statistically not significant (t=−99; df = 99; *p* = 0.84) in men. For women, the respective differences (M_1women_ = 18.74; 95% CI 17.78–19.71/ M_2women_ = 19.04; 95% CI: 18.07–20.01) were also not statistically significant (t = 0.53; df = 136; *p* = 0.29). In contrast, for vital body dynamics the scores (M_1men_ = 36.51; 95% CI 35.34–37.68/ M_2men_ = 34.11; 95% CI: 32.81–35.42) decreased over time in men (t = 4.14; df = 99; *p* < 0.001) and in women (M_1women_ = 36.42; 95% CI 35.47–37.37/ M_2women_ = 33.68; 95% CI 32.66–34.70; t = 5.84; df = 136; *p* < 0.001).

#### Effects of CHD-disease severity on body image (table 3)

In this latter line of analysis effects of disease severity on body image should be examined. For the first survey the regression analysis did not yield meaningful effects disease severity on “rejecting body evaluation”, but for vital body dynamics the effects were statistically significant and they were into the direction we had assumed. If compared with CHD-patients who underwent reparative surgery, the score of patients with reparative surgery was 2.40 points lower, and 3.77 points in patients with palliative surgery, thus indicating lower perceived physical performance with increasing disease severity. Sex and age turned out to be unrelated with variations of body image.

For the second survey, again no effects emerged for “rejecting body evaluation”, but for “vital body dynamics” decreasing perceived physical performance emerged with disease severity, but only for patients with palliative surgery the effect (4.08 points lower than in patients with curative surgery) was statistically significant. For this latter case an age effect was found, indicating lowering physical performance with increasing age.

**Table 3:** Effects of CHD-disease severity on body image with unstandardized regression coefficients, error levels and 95% confidence intervals after 200 bootstrap samples.

First survey						
	Rejecting body evaluation			Vital body dynamics		
	Coefficient	p	95% CI	Coefficient	p	95% CI
Disease severity, type of surgery						
Curative	1	–	–	1	–	–
Reparative	2.04	0.06	−0.05–4.12	−2.40	< 0.01	−4.11 – −0.70
Palliative	1.88	0.15	−0.67–4.44	−3.77	< 0.01	−6.49 – −1.06
Sex						
Male	1	–	–	1	–	–
Female	−1.57	0.05	−3.11–0.03	0.31	0.68	−1.21–1.83
Age	0.002	0.97	−0.095–0.099	0.016	0.71	−0.076–0.108
Constant	18.544	< 0.01	15.72–21.36	38.02	< 0.01	35.39–4.66

	Second survey					
	Rejecting body evaluation			Vital body dynamics		
	Coefficient	p	95% CI	Coefficient	p	95% CI
Disease severity, type of surgery						
Curative	1	–	–	1	–	–
Reparative	0.34	0.75	−1.78–2.47	−1.36	0.19	−3.37–0.67
Palliative	−0.39	0.77	−2.98–2.20	−4.08	< 0.01	−6.81 – −1.35
Sex						
Male	1	–	–	1	–	–
Female	−0.41	0.60	−1.96–1.13	0.10	0.90	−1.51–1.72
Age	−0.003	0.94	−0.093-0.086	−0.128	0.02	−0.236 – −0.019
Constant	19.45	< 0.01	15.68–23.22	40.27	< 0.01	36.34–44.20

## Discussion

With our analyses we wanted to examine body image as a consequence of congenital heart disease. Two dimensions of body image were considered as depicted by the FKB-20 as an instrument that was applied and tested in clinical as well as in non-clinical samples. We performed comparisons of CHD-patients with multivariately matched controls from the general population in order to examine disease-related specificities of body image as part of illness behaviour.

With respect to the first topic of study it turned out that in female CHD-patients both dimensions of body image did not differ from the general population. The situation in men was different as in both surveys they had consistently less favourable scores than men from the general population sample. Compared with women, male CHD-patients had a more negative body image and considered their body and the physical appearance as less good-looking. This critical attitude had also been found in an earlier and less elaborated analysis of data from the first survey^28^. Our findings are different from earlier studies based on psychopathological measures suggesting that women may be more affected than men^14^. In a study with *N* = 54 patients after Fontan surgery using a different measure of body image, women were reported to have a less favourable body image than male patients and controls from the general population^15^. There are however significant differences to our study. Besides a smaller sample, only patients who underwent one type of surgery were included, a different instrument for measuring body image was used, and controls were drawn from selective populations instead from the general population.

These findings do not explain why men had a less favourable body image than women as these findings are not in line with empirical studies from other areas of research^31,32^. There is evidence that in both sexes body image norms are conveyed via social comparison processes with peers^33^. In boys and male adults negative affect such as depression, anxiety and stress may play an import role^34^ for initiating activities to change body image. In a Finnish longitudinal study it was reported that men maintained a less positive body image over the life course, and it was less positive with decreasing socio-economic.

position^35^. This may be explained by a male emphasis for physical strength, and among male patients with congenital heart disease the difference between individual ability and what is considered as desirable may be particularly salient.

In the introduction we have noted that in earlier studies the psychopathological consequences of congenital heart disease had been examined. In our study we did not want to follow this line of research, and the decision to do so was made early when the study was planned. Comparisons of body image with other impairments and diseases had been performed^22,36,37^, , but none of them dealt with congenital heart disease.

In a more general perspective, the everyday life of patients with congenital heart disease appears as less severely impaired as it may seem. In a recent study with the same longitudinal dataset, we found no difference between patients and the general population in terms of success at school. Occupational careers also proceeded favourably, but a considerable proportion of patients retired prematurely or they never entered the labour force^18^. Selective participation is not a strong argument against these findings. Although patients with palliatively operated CHD were less likely to participate in the follow-up, this also holds for curatively treated patients^30^, probably due to lower frequencies of visits to the doctor resulting from lower attachment to the clinic where regular medical supervision is taking place.

The second topic of our study referred to the stability of the two dimensions of body image. Against the backdrop of significantly differing stability coefficients it turned out that both were of limited stability over time, although it has to be borne in mind that between both measurements about 15 years had elapsed. The stability coefficient of rejecting body evaluation was *r* = 0.34. However, as a considerable proportion of the patients were adolescents, changes of this dimension of body image is not only to be expected in patients with some defective appearance, but also in the adolescent general population^38,39^. Vitality as the dimension with closer relationship with everyday functioning turned out as more stable over time. This indicates that up to the age span covered in our study sample, up to the age of 59 patients were able to maintain their everyday functioning, although in an earlier publication it turned out that with increasing age participation in the labour force had decreased^18^, probably due to declining physical strength. This interpretation is supported by the statistically significant effect of age on vitality at follow-up.

The third topic of our study referred to effects of disease severity on the two dimensions of body image. Again, the findings differed by the dimension considered. For “rejecting body evaluation” significant regression effects were almost absent, thus supporting the interpretation that this dimension largely depicts subjective appraisal of physical appearance with small or no reference to other measures included in our study. For vitality the effects became stronger with increasing disease severity. If statistically significant, the unstandardized regression coefficients of vitality correspond to standardized effects reaching β *≥* 0.20, thus corresponding to moderate effect sizes. It has to be noted that for both dimensions it was largely irrelevant whether men or women were concerned.

Finally, some limitations of our study have to be admitted. Although we included a large sample, patients with palliatively operated CHD were underrepresented, and they were less likely to participate than reparatively operated patients^30^. This led to an underrepresentation of this group of patients and to more positive findings in analyses referring to the first and the second topic. Together with an underrepresentation of reparatively treated patients this might have led to underestimated effects and to more conservative interpretations. This is different from a Dutch study that reported higher proportions of patients with mild CHD, although the classifications were different from ours^11^. As a second limitation, the patients included in our study came from a single university medical centre. Although the clinic is specialized in the treatment of patients with congenital heart disease of all ages and has a large geographical catchment area, selection effects cannot be excluded. A third limitation refers to the control group from the general population. For both surveys we used data from a study that was conducted at the same time of our first survey. Comparisons with the first wave should thus not cause problems with the interpretation of our findings. We did not permit overlaps between controls drawn for the first and the second survey, but at the occasion of the second patient survey a longer time period had elapsed from the population study what leaves it open to what extent body image had changed in the meantime^34^. Nevertheless, we may claim that ours is the first study to having used multivariate matching at individual level with a random sample drawn from the general population in combination with a large patient sample and a long follow-up. The maximum age of CHD-patients was 59 years, and it may be expected that effects of disease severity will grow with increasing age. Although our study comprised a relatively large study sample that had not often been achieved in CHD-studies, case numbers have limited the depth of our statistical analyses. This applies to the models estimating effects of disease severity on body image as displayed in Table 3. Although reasonable, the available sample size did not permit interaction effects between disease severity and gender on body image.

Our study covered patients recruited in a time when survival was already good to very good for patients who underwent curative and palliative surgery. In the last decades survival rates had improved particularly in patients with complex and severe malformations^2^. Against the backdrop of this development it should be worth to examine how body image develops in a population of patients with severe CHD, where residual symptoms and impairments can be felt most of the time.

This leads to the question of potential interventions and support strategies that should be directed towards managing psychological concomitants of CHD and how to cope with limitations that may aggravate already relatively early in life, i.e. during adolescence. Whether such interventions are successful is questionable, and if they are, females may gain more than males^40^.

## Conclusions

Taking all the findings together, we may conclude that body image is a relevant psychological dimension not only relevant to adolescent patients with congenital heart disease, but also to adults. Contrary to expectation negative effects were mostly related to men what should draw the attention to the concomitants of their disease. Finally, vitality, but not rejecting body evaluation, was significantly associated with disease severity, thus pointing towards a realistic assessment of patients’ limited physical performance.

## Data Availability

The variables of the main dataset used for the current study are available from the corresponding author on reasonable request. The population data used for comparisons are available from EB on reasonable request (elmar.braehler@medizin.uni-leipzig.de).
